# Genetic variations, reproductive aging, and breast cancer risk in African American and European American women: The Women's Circle of Health Study

**DOI:** 10.1371/journal.pone.0187205

**Published:** 2017-10-26

**Authors:** Marie V. Coignet, Gary Robert Zirpoli, Michelle R. Roberts, Thaer Khoury, Elisa V. Bandera, Qianqian Zhu, Song Yao

**Affiliations:** 1 Department of Cancer Prevention and Control, Roswell Park Cancer Institute, Buffalo, NY, United States of America; 2 Department of Neurology, Massachusetts General Hospital, Harvard Medical School, Boston, MA, United States of America; 3 Channing Division of Network Medicine, Brigham and Women’s Hospital, Harvard Medical School, Boston, MA, United States of America; 4 Department of Pathology, Roswell Park Cancer Institute, Buffalo, NY, United States of America; 5 Cancer Prevention and Control Program, Rutgers Cancer Institute of New Jersey, New Brunswick, NJ, United States of America; 6 Department of Biostatistics, Roswell Park Cancer Institute, Buffalo, NY, United States of America; GeneDx, UNITED STATES

## Abstract

Reproductive aging phenotypes, including age at menarche (AM) and age at natural menopause (ANM), are well-established risk factors for breast cancer. In recent years, many genetic variants have been identified in association with AM and ANM in genome-wide association studies among European populations. Using data from the Women’s Circle of Health Study (WCHS) of 1,307 European-American (EA) and 1,365 African-American (AA) breast cancer cases and controls, we aimed to replicate 53 earlier GWAS variants for AM and ANM in AA and EA groups and to perform analyses on total and net reproductive lifespan (TRLS; NRLS). Breast cancer risk was also examined in relation to a polygenic risk score (PRS) for each of the reproductive aging phenotypes. We replicated a number of variants in EA women, including rs7759938 in *LIN28B* for AM and rs16991615 in *MCM8* for ANM; whereas in the AA group, only one SNP (rs2947411 in *TMEM18*) for AM was directionally consistent and nominally significant. In analysis of TRLS and NRLS, several SNPs were significant, including rs466639 in *RXRG* that was associated with both phenotypes in both AA and EA groups. None of the PRS was associated with breast cancer risk. Given the paucity of data available among AA populations, our study contributes to the literature of genetics of reproductive aging in AA women and highlights the importance of cross population replication of GWAS variants.

## Introduction

Menarche and menopause are two fundamental physiological events in a woman’s life, respectively marking the beginning and the end of reproductive age. At those turning points, estrogen milieu inside a woman’s body undergoes drastic changes, and it is the lifetime exposure to sex hormones that is long speculated to have a profound impact on women’s health, extended from reproduction to hormone-related morbidities including breast cancer. Early age at menarche (AM) and late age at natural menopause (ANM) are well-established risk factors for breast cancer. The risk was estimated to increase by a factor of 1.050 for each year earlier at menarche and by 1.029 for each year older at menopause[1].

As two complex numeric human traits, the timing of AM and ANM varies greatly among individuals. They are under the influence of an intricate set of social, environmental and genetic factors. It was estimated that genetic inheritance explains over half of the variation (heritability for AM at 53–74% and for ANM at 44–65%)[2–9], which provides a strong rationale for genetic association studies to identify genes and variants determining their timing. To date, at least 17 genome-wide association studies (GWAS) have been conducted on AM and ANM[10–27], which have discovered a large number of common (minor allele frequency >5%) variants reliably associated with each of them. Results from these studies suggest that the genetic architecture of AM and ANM is polygenic, involving hundreds of variants, each with small effect, in multiple genes and biological pathways. It is worth noting that AM and ANM are typically studied separated and few studies have examined both in the same population; thus, it is largely unclear to what extent those two reproductive aging phenotypes share genetic determinants.

Racial/ethnic minority populations, particularly African Americans (AA), are under-represented in the GWAS of reproductive aging phenotypes in previous studies. Among the published GWAS, only one on AM[22] and one on ANM[23] were conducted among AA women. There are at least two reasons that more research efforts are warranted in genomic studies in minority populations. First, given the racial/ethnic diversity in genetic background and possibly in pathogenesis of cancer, it is not only prudent but imperative to replicate variants initially identified from populations of European ancestry (EA) in racial minorities, before the findings can be generalized across populations[28]. Second, AA women have distinct reproductive profiles, compared to EA women; for example, it has been shown that compared to EAs, AA women experience an earlier AM[29–31] as well as an earlier ANM[32, 33]. It is thus possible that the genetic architecture underlying reproductive aging phenotypes is distinct in AA populations. Further, reproductive factors have been postulated as important contributors to breast cancer health disparities between AA and EA women, including an earlier age at diagnosis and more aggressive pathological presentation in the former. Two recent studies show evidence that genetic variants associated with AM are also associated with breast cancer risk[27, 34]. It yet remains to be explored whether those variants contribute to the health disparities.

In the Women’s Circle of Health Study (WCHS), which enrolled a similar number of AA and EA women, with and without breast cancer, we aimed to replicate earlier GWAS findings in both AA and EA women, as well as to examine them with breast cancer risk in the two groups.

## Methods

### Study population

The WCHS was designed to study genetic and non-genetic factors in association with early and aggressive breast cancer risk in EA and AA women. A more detailed description of the study population has been published elsewhere[35–38]. In brief, case ascertainment in early years of the study was conducted through hospital-based referrals at targeted hospitals with large AA patient populations in 4 boroughs of New York City, and later shifted to population-based in 7 counties of New Jersey through the NJ State Cancer Registry. Case eligibility was determined by being a self-identified AA or EA woman, English speaking, between 20–75 years of age at diagnosis of primary, histologically confirmed breast cancer, with no history of cancer except for non-melanoma skin cancer. Since AA women are more likely to be diagnosed with breast cancer at an earlier age, younger women were oversampled in the enrollment. As a result, the population was younger relative to many other studies of breast cancer. Controls with no prior cancer history and matched to the cases by self-reported race, 5 year age categories and geographic residence were initially identified through random digit dialing (RDD), and later through community recruitment, especially to boost enrollment of AA controls. In a methodological analysis in the WCHS, a combination of community controls and RDD controls were found to be better representative of the general population than RDD controls alone[39]. Written informed consent was obtained from all participants and an in-person interview was administered to collect a wide range of epidemiological data, anthropometric measures and biospecimens. Blood samples were initially collected and later switched to saliva as a source of genomic DNA. The informed consent included permission to request pathology data and tumor tissue blocks and slides from attending hospitals. This study was approved by the Institutional Review Boards at Roswell Park Cancer Institute (RPCI), Rutgers Cancer Institute of New Jersey (CINJ), Icahn School of Medicine at Mount Sinai, and the participating hospitals in NYC.

### SNP selection and genotyping

Our goal was to replicate single nucleotide polymorphisms (SNPs) that were previously identified in GWAS of AM and ANM in EA populations. Therefore, we selected 53 SNPs from 5 GWAS published by June 2011[13, 16, 24–26] when a custom genotyping chip was designed. These include 41 AM SNPs and 12 ANM SNP. Detailed information of the selected SNPs is summarized in S1 Table. Genotyping was attempted for 2,762 participants who had been enrolled into the WCHS and had genomic DNA samples available at that time, using the Illumina GoldenGate assay (Illumina Inc., San Diego, CA) performed by the Genomics Shared Resource at Roswell Park Cancer Institute. The average per-sample successful genotyping rate was 95.9%; 38 samples had calls less than 90% and were excluded. Quality control samples consisted of 5% blind duplicates and two sets of in-house trio samples. Concordance was >99.9% between duplicate pairs and there were no Mendelian errors in trio control samples. No SNPs selected for this analysis had a successful call rate <90%, or violated Hardy Weinberg Equilibrium when checked separately in AA and EA controls. SNPs with significant associations identified in the analyses were manually checked *post hoc* for the clustering plots, to ensure call robustness.

### Genetic ancestry estimation

A panel of ancestry informative markers (AIMs) developed by the Black Women’s Health Study was included as part of the customized genotyping array and the data were used to ascertain genetic ancestry and to control for population admixture[40]. Estimates of the proportion of European and African ancestry were obtained using the STRUCTURE program[41]. Individuals (n = 11 in AAs and n = 1 in EAs) with a greater than 85% estimated ancestry that was discordant with the self-reported ancestry were excluded from analyses. The estimated percent European genetic ancestry was included as a numeric confounder in multivariate models.

### Statistical analysis

The final dataset included 2,672 women, consisting of 658 EA breast cancer cases, 649 EA controls, 621 AA cases, and 744 AA controls. All analyses were performed separately for AA and EA women. T-tests or chi-square tests were used to compare descriptive characteristics between breast cancer cases and controls in univariate analyses. The two primary phenotypes, AM and ANM, were obtained via self-reported menstrual and pregnancy history. Women were defined as postmenopausal if they reported that they had ceased menstruation naturally for at least 12 consecutive months and those who had hysterectomy or oophorectomy were excluded from analysis of ANM, TRLS, and NRLS (n = 356). In addition to AM and ANM, we also computed another two phenotypes, total reproductive lifespan (TRLS) and and net reproductive lifespan (NRLS), using the numeric variables of AM and ANM. We considered TRLS to be the difference between ANM and AM, and NRLS to be equal to TRLS with time spent on all pregnancies and breastfeeding subtracted. Except for AM, the other three phenotypes were analyzed only among postmenopausal women. For AM analyses, 1365 AA women and 1307 EA women contributed in these analyses. For ANM/TRLS/NRLS analyses, 381 AA women and 465 EA women were included in the analyses.

Each single SNP was related to one of the four reproductive aging phenotypes, AM, ANM, TRLS and NRLS, using generalized linear modeling (GLM) with adjustment chosen a priori for birth year, estimated proportion of European ancestry, smoking (not for AM), and number of pregnancies (not for AM or NRLS) ensuring a parsimonious model. Additive genetic models were tested, with the common allele in the EA group designated for both AA and EA groups as the reference allele. Genotype analyses were first conducted in the case and control groups separately, which produced estimates of similar magnitude in the same direction. Thus, all analyses were conducted with case-control combined and the case-control status was included as a covariate in the GLMs. For the purpose of our study, replication of previous GWAS hits was defined by nominal significance of p≤0.05 and concordance in direction of effect for the same phenotype.

To test whether SNPs associated with reproductive aging had an impact on breast cancer risk, a polygenic risk score (PRS) was calculated for each of the four reproductive phenotypes, combining the effects of all SNPs associated with the phenotype at a nominal p-value <0.10. PRS for each phenotype and for each individual was calculated as a sum of the number of risk alleles, weighted by the regression coefficients from GLM. To align SNPs with opposite directions in their associations with a phenotype, the reference allele of those with a negative regression coefficient was flipped, so the regression coefficient became positive and could be tallied with other SNPs. As a result, a higher PRS indicated an older AM or ANM, or a longer TRLS and NRLS. Each PRS was then categorized into quartiles based on the distribution among controls, and related to breast cancer case-control status with a priori adjustment for age, estimated proportion of European ancestry, and family history of breast cancer, ensuring a parsimonious model. All analyses were performed using SAS 9.4 (SAS Institute, Cary, NC).

## Results

### Descriptive characteristics of the study population

Table 1 summarizes selected descriptive characteristics of the study population by self-reported race. Because of the oversampling of younger women, more than half of the study population was premenopausal. Compared to EA women, AA women were slightly younger at enrollment, had a significantly higher BMI and lower education attainment, and were less likely to be a never smoker and to have family history of breast cancer. For reproductive aging phenotypes, AA women had a similar AM, but younger ANM and shorter TRLS and NRLS than EA women. Further comparisons of the descriptive characteristics between breast cancer cases and controls within each racial group are shown in S2 Table. In both AA and EA groups, there were no significant differences in AM or ANM between cases and controls; whereas cases had longer TRLS and NRLS than controls (p≤0.016 except for NRLS in AAs).

**Table 1:** Descriptive characteristics of the Women’s Circle of Health Study.

Characteristics	African American (n = 1365)	European American (n = 1307)	P-value*
Age, years, mean (range)	49.9 (22–75)	50.9 (26–75)	0.009
Current body mass index, kg/m^2^, mean (SD)	31.5 (7.3)	27.25 (6.8)	< .0001
Percent European Ancestry, mean (SD)	13.7 (0.15)	97.6 (0.06)	< .0001
Education, n(%)			< .0001
High school or less	575 (42.1)	208 (15.9)	
College	628 (46.0)	672 (51.4)	
Post-graduate degree	162 (11.9)	427 (32.7)	
Smoking status, n (%)			< .0001
Never smoker	814 (59.6)	701 (53.6)	
Former smoker	288 (21.1)	449 (34.4)	
Current smoker	263 (19.3)	156 (11.9)	
Family history of breast cancer, n(%)			< .0001
Yes	176 (12.9)	270 (20.7)	
No	1189 (87.1)	1037 (79.3)	
Age at menarche, years, mean (SD)	12.5 (1.8)	12.6 (1.6)	0.51
Age at menarche, n (%)			0.27
≤ 12 years of age	698 (51.2)	635 (48.9)	
> 12 years of age	666 (48.8)	662 (51.1)	
Menopause status, n (%)			0.76
Premenopausal	721 (52.8)	699 (53.5)	
Postmenopausal	644 (47.2)	608 (46.5)	
Age at menopause, years, mean (SD)	49.8 (4.9)	50.2 (4.2)	0.198
Age at menopause, n (%)			0.283
≤ 50 years of age	190 (51.4)	214 (47.3)	
> 50 years of age	180 (48.6)	238 (52.7)	
Total reproductive life span, years, mean (SD)	37.2 (5.5)	37.8 (4.3)	0.09
Net reproductive life span, years, mean (SD)	34.6 (6.1)	35.9 (4.7)	0.0003

SD, standard deviation.

* P-value from student's T-test or chi-square test.

### Age at menarche (AM)

In EA women, 31 out of 37 SNPs selected for association with AM were in the same direction as in the previous GWAS (p = 3.7e-6, binomial sign test), including 4 with a nominal significance of p≤0.05 and another 5 with a suggestive significance of p≤0.10 (Table 2). The top SNP, rs7759938 in *LIN28B*, was one of the most consistent variants in previous GWAS for AM. In our study, rs7759938 was associated with a 0.2 year of delay in menarche per copy of the variant G allele (p = 0.004). In addition, rs4843747 in 16q24.2–3, which was associated with later ANM in a previous GWAS, was shown to be significantly associated with a 0.2-year delay in AM per copy of the variant A allele (p = 0.01) in EA women in our study. In AA women, 20 out of 37 SNPs selected for association with AM were in the same direction as in the previous GWAS (p = 0.37, binomial sign test); yet only rs2947411 in *TMEM18* reached a nominal significance (p = 0.03) in our study. rs757647 in *KDM3B*, which was associated with earlier AM in EA women in our study, was also significant in AA women but in an opposite direction.

**Table 2:** Top-ranked SNPs associated with age at menarche (p≤0.10).

Race	Gene/Region	SNP	Chr	Coordinate	Alleles	MAF	Common homozygote Mean±SD	Heterozygote Mean±SD	Rare homozygote Mean±SD	β±SE per copy variant allele, years*	P-value*
AA	11q25	rs4397868	11	133566985	G/A	0.04	12.6±1.8	12.0±1.8	9.7±1.2	-0.60±0.18	0.001
	KDM3B	rs757647	5	137707315	A/G	0.42	12.3±1.8	12.6±1.8	12.8±2	0.21±0.07	0.002
	TMEM18	rs2947411	2	614168	A/G	0.23	12.4±1.8	12.6±1.9	12.9±1.8	0.18±0.08	0.03
	2q24.2–3	rs11889862	2	150697148	A/G	0.32	12.6±1.8	12.5±1.9	12.2±1.7	-0.15±0.07	0.05
	TRMT6	rs16991615	20	5948227	A/G	0.01	12.5±1.8	13.1±2	N/A	0.75±0.38	0.05
EA	LIN28B	rs7759938	6	105378954	G/A	0.3	12.4±1.5	12.7±1.5	12.6±1.6	0.19±0.07	0.004
	RXRG	rs466639	1	165394882	A/G	0.11	12.6±1.6	12.4±1.4	12.3±2	-0.26±0.1	0.008
	16q24.2–3	rs4843747	16	87991051	A/C	0.17	12.5±1.6	12.7±1.5	12.9±1.9	0.2±0.08	0.01
	VGLL3	rs7642134	3	86916882	A/G	0.38	12.7±1.6	12.5±1.5	12.4±1.7	-0.14±0.06	0.03
	PXMP3	rs7821178	8	78093837	A/C	0.34	12.6±1.6	12.6±1.5	12.3±1.6	-0.13±0.06	0.05
	9q31.2	rs7861820	9	108936674	A/G	0.48	12.4±1.6	12.6±1.5	12.7±1.6	0.12±0.06	0.06
	INHBA	rs1079866	7	41470093	G/C	0.15	12.5±1.5	12.6±1.5	13±1.6	0.15±0.08	0.06
	KLHDC8B	rs7617480	3	49210732	A/C	0.24	12.5±1.6	12.5±1.5	13±1.6	0.13±0.07	0.06
	ARHGEF7	rs9555810	13	112181437	G/C	0.28	12.5±1.5	12.6±1.6	12.8±1.6	0.12±0.07	0.08
	KDM3B	rs757647	5	137707315	A/G	0.25	12.6±1.6	12.5±1.5	12.3±1.5	-0.12±0.07	0.08

MAF, minor allele frequency; SD: standard deviation; SE: standard error.

*Linear regression coefficients (β) and P-value adjusted for genetic ancestry, year of birth, and breast cancer case-control status.

### Age at natural menopause (ANM)

In EA women, 2 out of 16 SNPs selected for association with ANM were in the same direction as in the previous GWAS and reached a nominal significance of p≤0.05 (Table 3). The top SNP, rs16991615 in *MCM8* was also one of the most significant SNPs identified in GWAS for ANM, with each copy of the variant A allele associated with 1.3-year delay in the onset of menopause. rs236114, another SNP in *MCM8*, was also replicated with ANM in EA women in our study with a suggestive level of significance (p = 0.08). In addition, two SNPs previously associated with AM, rs7821178 in *PXMP3* and rs6589964 in *BSX*, were associated with ANM in EA women in our study. In AA women, none of the selected SNPs for association with ANM were in the same direction and reached a nominal significance. Instead, five SNPs previously associated with AM were significant with a nominal p≤0.10, including rs1859345 in *SPOCK*.

**Table 3:** Top-ranked SNPs associated with age at menopause (p≤0.10).

Race	Gene/Region	SNP	Chr	Coordinate	Alleles	MAF	Common homozygote Mean±SD	Heterozygote Mean±SD	Rare homozygote Mean±SD	β±SE per copy variant allele, years*	P-value*
AA	SPOCK	rs1859345	5	136447420	G/A	0.13	50.3±4.7	48.6±5.4	50±4.2	-1.55±0.54	0.004
	LIN28B	rs314280	6	105400837	A/G	0.75	48.3±5.4	49.5±4.5	50.3±5	0.78±0.4	0.05
	PHF21A	rs16938437	11	46052575	A/G	0.23	50.2±4.4	49.7±5.2	47.7±6.6	-0.68±0.39	0.09
	PLCL1	rs12617311	2	199632565	A/G	0.14	50.1±4.8	49.1±5.1	50.6±3.9	-0.81±0.49	0.10
	RXRG	rs466639	1	165394882	A/G	0.14	49.8±5.1	50±4.4	51.8±1.5	0.84±0.51	0.10
EA	MCM8	rs16991615	20	5948227	A/G	0.08	50.1±4.4	51.5±3.2	52.9±2.8	1.29±0.47	0.006
	PXMP3	rs7821178	8	78093837	A/C	0.35	50.6±3.8	50.2±4.3	49.3±5.3	-0.66±0.28	0.02
	BRSK1	rs1172822	19	55819845	A/G	0.37	50.7±4.1	50.2±4.2	49.3±4.6	-0.59±0.28	0.04
	MCM8	rs236114	20	5935385	A/G	0.22	50±4.3	50.6±4.2	51.8±3.2	0.58±0.32	0.08
	BSX	rs6589964	11	122870683	A/C	0.44	50.8±4.6	50.2±4	49.7±4.1	-0.47±0.27	0.08

*Linear regression coefficients (β) and P-value adjusted for genetic ancestry, year of birth, smoking, number of full-term pregnancy, and breast cancer case-control status.

MAF, minor allele frequency; SD: standard deviation; SE: standard error.

### Total reproductive lifespan (TRLS) and net reproductive lifespan (NRLS)

Tables 4 and 5 present the top ranked SNPs associated with TRLS and NRLS, respectively. It is noteworthy that a majority of those SNPs were associated with either AM or ANM in our study as described above, and most of them were associated with both TRLS and NRLS. The top SNP associated with TRLS was rs16991615 in *MCM8* in EA women and rs1859345 in *SPOCK* in AA women, both of which were also associated with ANM and NRLS. Their effect sizes were similar across the three phenotypes (1.1 to 1.3 years per copy of the variant A allele for rs16991615 and -1.6 to -1.7 years per copy of the variant G allele). The top SNP associated with NRLS in EA was rs1079866 in *INHBA*, which was also associated with AM in our analysis. Per copy of the variant G allele was associated with a 0.9 year shorter in NRLS, whereas it was associated with only a 0.2-year delay in AM. Another noteworthy SNP is rs466639 in *RXRG*, which was associated with both TRLS and NRLS in both AA and EA women. Its effect size appeared to be slightly larger for NRLS (1.1 to 1.4 years per copy of the variant A allele) than for TRLS (0.8 to 1.0 year), the latter of which was similar to its effect size for ANM in AAs (0.8 year per copy).

**Table 4:** Top-ranked SNPs associated with total reproductive lifespan (p≤0.10).

Race	Gene/Region	SNP	Chr	Coordinate	Alleles	MAF	Common homozygote Mean±SD	Heterozygote Mean±SD	Rare homozygote Mean±SD	β±SE per copy variant allele, years*	P-value*
AA	SPOCK	rs1859345	5	136447420	G/A	0.13	37.6±5.3	35.9±5.9	38±2.8	-1.59±0.61	0.009
	PHF21A	rs16938437	11	46052575	A/G	0.23	37.5±5.1	37.1±5.8	34.2±6.4	-0.88±0.45	0.05
	LIN28B	rs314280	6	105400837	A/G	0.75	35.7±5.7	36.8±5.2	37.6±5.6	0.8±0.45	0.08
	RXRG	rs466639	1	165394882	A/G	0.14	37.1±5.6	37.3±5.2	39.9±2.1	0.99±0.58	0.09
EA	MCM8	rs16991615	20	5948227	A/G	0.08	37.5±4.5	39±3.4	40.1±3.2	1.24±0.48	0.01
	6p21.3	rs494620	6	31838713	G/A	0.49	37.9±4.5	38.2±3.6	36.8±5.2	-0.53±0.27	0.05
	BRSK1	rs1172822	19	55819845	A/G	0.37	38.2±4.4	37.7±4.2	36.8±4.7	-0.57±0.29	0.05
	RXRG	rs466639	1	165394882	A/G	0.11	37.6±4.5	38.4±3.9	39.3±1.7	0.79±0.46	0.09
	PRDM13	rs4840086	6	100208438	G/A	0.45	37±4.7	38.5±4	37.4±4.5	0.46±0.28	0.10

MAF, minor allele frequency; SD: standard deviation; SE: standard error.

*Linear regression coefficients (β) and P-value adjusted for genetic ancestry, year of birth, smoking, number of full-term pregnancy, and breast cancer case-control status.

**Table 5:** Top-ranked SNPs associated with net reproductive lifespan (p≤0.10).

Race	Gene/Region	SNP	Chr	Coordinate	Alleles	MAF	Common homozygote Mean±SD	Heterozygote Mean±SD	Rare homozygote Mean±SD	β±SE per copy variant allele, years*	P-value*
AA	SPOCK	rs1859345	5	136447420	G/A	0.13	34.9±6	33.1±6.2	36.9±1.2	-1.73±0.69	0.01
	LIN28B	rs314280	6	105400837	A/G	0.75	32.3±7	34.4±5.8	34.9±6.2	1.12±0.52	0.03
	RXRG	rs466639	1	165394882	A/G	0.14	34.3±6.3	35±5.4	37.6±3.3	1.4±0.66	0.03
	PHF15	rs13187289	5	133849177	C/G	0.21	34.3±6.3	34.8±5.6	38.8±3.9	1.08±0.56	0.05
	PHF21A	rs16938437	11	46052575	A/G	0.23	34.9±5.7	34.4±6.4	31.6±7.5	-0.9±0.51	0.08
EA	INHBA	rs1079866	7	41470093	G/C	0.15	36.2±4.6	35.7±4.9	33.3±5	-0.93±0.41	0.02
	RXRG	rs466639	1	165394882	A/G	0.11	35.7±4.9	36.8±4	38.6±1.3	1.11±0.5	0.03
	MCM8	rs16991615	20	5948227	A/G	0.08	35.8±4.8	36.9±4.1	38.9±3.4	1.1±0.53	0.04
	6p21.3	rs494620	6	31838713	G/A	0.49	36.2±4.7	36.4±4.1	35±5.5	-0.6±0.29	0.04

MAF, minor allele frequency; SD: standard deviation; SE: standard error.

*Linear regression coefficients (β) and P-value adjusted for genetic ancestry, year of birth, smoking, and breast cancer case-control status.

### Polygenic risk scores (PRS) for reproductive aging phenotypes and breast cancer risk

The correlations between the PRS for each reproductive aging phenotype and the phenotype itself were highly significant as expected (p≤0.0002); yet the strength of the correlations was weak (correlation coefficient 0.16–0.21) (Table 6). None of the PRS was in significant association with breast cancer risk in either AA or EA women, with the exception that the third highest quartile of the PRS for NRSL was associated with an increased risk compared to the lowest quartile (odds ratio 2.19, 95% confident interval 1.14–4.23). Nevertheless, the trend test was not statistically significant (p = 0.10).

**Table 6:** Associations of polygenic scores of reproductive aging and breast cancer risk.

Reproductive aging phenotype	Race	Correlation coefficient*	P-value	Odds ratio (95% confidence interval)				P-value^†^
				Q1	Q2	Q3	Q4	
Age at menarche	AA	0.16	< .0001	1.00	1.28 (0.93–1.77)	0.94 (0.71–1.24)	1.06 (0.78–1.43)	0.28
	EA	0.19	< .0001	1.00	0.85 (0.62–1.16)	0.83 (0.61–1.14)	0.85 (0.63–1.14)	0.61
Age at natural menopause	AA	0.21	< .0001	1.00	1.34 (0.71–2.52)	1.39 (0.73–2.63)	1.18 (0.63–2.21)	0.74
	EA	0.21	< .0001	1.00	0.94 (0.55–1.60)	0.53 (0.29–0.94)	0.78 (0.46–1.32)	0.14
Total reproductive life span	AA	0.20	0.0002	1.00	1.39 (0.73–2.65)	1.94 (0.97–3.89)	1.48 (0.80–2.73)	0.31
	EA	0.19	< .0001	1.00	0.54 (0.31–0.96)	0.76 (0.44–1.32)	0.65 (0.37–1.13)	0.18
Net reproductive life span	AA	0.21	< .0001	1.00	1.46 (0.75–2.82)	2.19 (1.14–4.23)	1.32 (0.68–2.59)	0.10
	EA	0.19	< .0001	1.00	0.67 (0.27–1.66)	0.65 (0.39–1.09)	0.87 (0.53–1.43)	0.39

*Correlation coefficient of the polygenic scores of reproductive aging phenotype with the corresponding phenotype.

† p-value adjusted for index age, genetic ancestry and family history of breast cancer.

## Discussion

In a breast cancer case-control study with similar number of AA and EA women, we replicated several SNPs identified in previous GWAS of AM and ANM in the EA group, including the top GWAS signals rs7759938 in *LIN28B* and rs16991615 in *MCM8*. Nevertheless, the replication of the same set of SNPs in AA women was not as successful. Only one out of the 37 selected SNPs for AM was significant in the same direction, and none of the 16 selected SNPs for ANM was significant. In analyses of TRLS and NRLS, a number of SNPs associated with AN or ANM in previous GWAS were associated with the two reproductive lifespan phenotypes, and most of the associations were consistent between the two phenotypes with similar effect sizes. We found little evidence for a strong impact of those reproductive aging-associated SNPs on breast cancer risk. Our analysis suggests that the overlap of genetic determinants of reproductive aging phenotypes between AA and EA populations may be limited, and highlights the importance of evaluating GWAS signals initially identified from EA populations in AA populations, before they can be generalized across populations.

In the past decade, GWAS has been a powerhouse in discovering common genetic variants reliably associated with a wide range of complex phenotypes and common diseases. To date, the only GWAS on AM and ANM among AA women did not identify any variants at a genome-wide significant level (5e-8)[22, 23], which is a striking difference from the remarkable success of similar studies conducted among populations of European descent. An apparent reason is the scarcity of large epidemiological populations of African descent. Another reason may be related to their distinct genetic architecture. Compared to Europeans and East Asians, African populations have more unique genetic variations and shorter linkage disequilibrium (LD) blocks across the genome, features that require GWAS in African populations to have larger sample sizes. Due to these limitations, replication studies provide a feasible alternative approach to evaluate previous EA GWAS hits in people of African ancestry. Yet, in studies among AA women in the Population Architecture Using Genomics and Epidemiology (PAGE) Study, the generalization of the previously identified GWAS variants, including rs7759938 in *LIN28B* for AM and rs16991615 in *MCM8* for ANM, was mostly unsuccessful[10, 21]. This is consistent with our data, where only one out of 37 SNPs for AM and none out of 16 SNPs for ANM was replicated in AA women. It should be noted that rs7759938 was associated with AM in AA women in a replication analysis nested in a previous GWAS[22], but it was not significant in either our study or in PAGE. The lack of generalization of GWAS variants for AM and ANM in AAs is in contrast to similar studies in East Asians and Hispanics, where a majority of the variants evaluated were replicated[42–44]. Considering the wide difference in allele frequency and LD between European and African populations, it is possible that genes or loci associated with AM and ANM are the same between the two populations, but index SNPs that are mostly likely to be identified in association studies may be different. Fine-mapping analyses of the entire regions of the previously identified loci, but not only the index SNPs, in the two GWAS among AA women provide some support for this hypothesis [22, 23]. However, the fact that none of the variants in AAs met the commonly accepted threshold for genome-wide significance calls for definitive evidence from future studies based on larger sample sizes.

Although most of the identified associations with AM and ANM in our study did not overlap between AA and EA women, a phenomenon we have repeatedly observed in previous studies of breast cancer risk[35–37, 45, 46], we found that rs466639 in *RXRG* was associated with TRLS and NRLS in both AA and EA women with a similar effect size. This SNP was initially identified in association with AM in Europeans, with an effect size of 4.2 months earlier in AM per copy of the variant T allele[25]. We replicated the association of this SNP with AM in EA women. Although it was not associated with AM in AA women, this SNP was associated with a delay in ANM in AA women but not in EA women. It is thus possible that its association with reproductive lifespan was driven by the effect on AM in EAs and the effect on ANM in AAs. This SNP is intronic in *RXRG*, which encodes retinoid X receptor gamma, a nuclear receptor that dimerizes with the receptors for retinoic acid, thyroid hormone and vitamin D and increases their DNA binding and transcriptional activity[47]. However, the biological impact of the SNP on gene expression and the mechanism of RXRG in regulating reproductive aging, remain unclear. In a recent study, rs466639 was associated with involution of terminal duct lobular unit (TDLU), the milk-producing structures of the breast[48]. The variant T allele associated with earlier AM was also associated with a higher count of TDLU, which is a recognized risk factor for breast cancer. In another study of AM SNPs with pubertal growth in male and female adolescents, rs466639 was not associated with changes in weight, height or BMI; nevertheless, four other AM SNPs were, suggesting shared genetic influences on onset of menstrual cycle and pubertal body growth[49].

To our knowledge, the literature on the overlapping of genetic underpinning between AM and ANM has been rather limited. In an early study in 1998 by Sneider et al., no correlation was found between age at menopause and age at menarche, suggesting distinct genetic mechanisms for the two phenotypes.[6] In another study by Spencer et al., two genes, ARHGAP42 and PHACTR1, which were associated with both AM and ANM; nevertheless, the associated SNPs within each gene were different between the two phenotypes. Although the directions of the associations were similar, those SNPs were not in strong LD.[21] Therefore, current data appear not to support large overlapping of genetic determinants for AM and ANM. Although the reasons are unknown, it could be due to the fact that the occurrence of ANM has a much longer time window susceptible to impact of various environmental factors in comparison to AM, and thus the identification of genetic determinants could be more difficult for the former. It may be interesting to note that far more GWAS have been done for AM than ANM. Clearly, future genetic association studies are warranted, especially for ANM.

Given the well-established associations of early AM and late ANM with breast cancer risk, it is a reasonable next step to examine whether GWAS SNPs for reproductive aging are associated with breast cancer risk, an approach known as Mendelian randomization analysis that is less likely to be biased by confounding factors and can be used to infer causality. In the first Mendelian randomization analysis for breast cancer risk, He and colleagues examined 19 AM SNPs and 17 ANM SNPs, individually and as PRS for early AM to combine the effects of multiple markers, in the ReproGen Consortium[11]. Two AM SNPs and one ANM SNP were associated with breast cancer in the expected direction. In addition, the 4^th^ and 5^th^ highest quintiles of PRS for AM were associated with increased breast cancer risk, with a borderline significant trend; yet no association was found with PRS for ANM. In a recent large study of PRS based on 375 autosomal AM variants, increasing AM was associated with lower risk of breast cancer, particularly estrogen receptor (ER) positive cancer, as well as ovarian cancer and endometrial cancer, which was independent of the effects of those variants on obesity[50]. The lack of associations of PRS for reproductive aging phenotypes with breast cancer risk in either AA or EA women in our study could be due to the limited number of GWAS variants included in our study. As a result, the proportion of phenotypic variations captured by those SNPs is likely low, and their effects on breast cancer risk might be too modest to be detected with our sample size.

We noted a few limitations in our study. Because genotyping was performed in 2011, which predated a number of large-scale GWAS for AM and ANM, our analysis included only variants identified from earlier studies and missed a substantial proportion of variants known to date. The sample size was also limited, especially for analysis of phenotypes among postmenopausal women. In addition, none of the SNPs for the four phenotypes passed the significance threshold (*α* = 0.05) after controlling for multiple comparisons via FDR correction. Therefore, our results should be interpreted with caution of small sample size. This is particularly relevant to our speculation of different genetic underpinning for AM and ANM based on the marked lower replication success rate in AAs than in EAs. Due to limited statistical power, we evaluated the concordance of the direction of associations between our study and previous GWAS literature, as an additional criterion for replication success, which is not stringent statistically speaking. However, our study is among the first to include both AA and EA women from a well-characterized study for breast cancer health disparities. Given the challenges in conducting fully powered GWAS among AA populations and the paucity of data available, our study contributes to the literature for genetics of reproductive aging phenotypes in AA women and highlights the importance of cross-race/ethnicity replication of GWAS variants. Another strength is the inclusion of reproductive lifespans in our analysis, which may have a higher impact on the risk of breast cancer than either AM or ANM alone.

In conclusion, in the WCHS, we replicated several GWAS variants for AM and ANM in EA women, but few were replicated in AA women. We identified rs466639 in *RXRG* associated with TRLS and NRLS in both AA and EA groups. However, none of the PRS for reproductive aging phenotypes were in significant association with breast cancer risk, which could be due to the limited number of GWAS variants included in our study, small sample size, and possibly low power. Future studies are warranted to investigate whether differences in reproductive aging-related genetic variants explain health disparities in breast cancer.

## Supporting information

S1 Table Summary of detailed information on selected SNPs. (PDF)

S2 Table Descriptive characteristics of the Women's Circle of Health Study. (PDF)
