# Supplementary material for: Genetic variations, reproductive aging, and breast cancer risk in African American and European American women: The Women's Circle of Health Study
Source: PLoS One. 2017 Oct 26;12(10):e0187205. doi: 10.1371/journal.pone.0187205 (PMC5658184; doi:10.1371/journal.pone.0187205)
Supplement: S1 Table — (PDF) [file pone.0187205.s001.pdf]

**S1 Table. Summary of detailed information on selected SNPs.**

| Gene/Region | SNP | Chr | Coordinate | Alleles | MAF | Ref(PMID) | Pheno_Lit |
|---|---|---|---|---|---|---|---|
| PRDM13 | rs4840086 | 6 | 100208438 | G/A | 0.11 | 21102462 | AM |
| ARHGEF7 | rs9555810 | 13 | 112181437 | G/C | 0.2 | 21102462 | AM |
| ARNTL | rs900145 | 11 | 13293905 | G/A | 0.56 | 21102462 | AM |
| BEGAIN | rs6575793 | 14 | 101032217 | G/A | 0.8 | 21102462 | AM |
| BSX | rs6589964 | 11 | 122870683 | A/C | 0.38 | 21102462 | AM |
| CA10 | rs9635759 | 17 | 49613785 | A/G | 0.13 | 21102462 | AM |
| CCDC85A | rs17268785 | 2 | 56592083 | G/A | 0.23 | 21102462 | AM |
| EEFSEC | rs2687729 | 3 | 127895226 | G/A | 0.33 | 21102462 | AM |
| FUSSEL18 | rs1398217 | 18 | 44752238 | G/C | 0.23 | 21102462 | AM |
| GAB2 | rs10899489 | 11 | 78095373 | A/C | 0.32 | 21102462 | AM |
| INHBA | rs1079866 | 7 | 41470093 | G/C | 0.09 | 21102462 | AM |
| Intergenic | rs7861820 | 9 | 108936674 | A/G | 0.1 | 19448621 | AM |
| IQCH | rs7359257 | 15 | 67702907 | A/C | 0.35 | 21102462 | AM |
| KDM3B | rs757647 | 5 | 137707315 | A/G | 0.42 | 21102462 | AM |
| KLHDC8B | rs7617480 | 3 | 49210732 | A/C | 0.3 | 21102462 | AM |
| LIN28B | rs314280 | 6 | 105400837 | A/G | 0.76 | 19448621 | AM |
| LIN28B | rs7759938 | 6 | 105378954 | G/A | 0.54 | 21102462 | AM |

| | | | | | | | |
|---|---|---|---|---|---|---|---|
| LRP1B | rs12472911 | 2 | 142228509 | G/A | 0.54 | 21102462 | AM |
| MKL2 | rs1659127 | 16 | 14388305 | A/G | 0.3 | 21102462 | AM |
| NFAT5 | rs1364063 | 16 | 69588572 | G/A | 0.24 | 21102462 | AM |
| NPHP3 | rs6439371 | 3 | 132610752 | G/A | 0.39 | 21102462 | AM |
| NR4A2 | rs17188434 | 2 | 157096776 | G/A | 0.01 | 21102462 | AM |
| OLFM2 | rs1862471 | 19 | 10000322 | C/G | 0.2 | 21102462 | AM |
| PCSK2 | rs852069 | 20 | 17122593 | A/G | 0.49 | 21102462 | AM |
| PHF15 | rs13187289 | 5 | 133849177 | C/G | 0.21 | 21102462 | AM |
| PHF21A | rs16938437 | 11 | 46052575 | A/G | 0.25 | 21102462 | AM |
| PLCL1 | rs12617311 | 2 | 199632565 | A/G | 0.15 | 21102462 | AM |
| PXMP3 | rs7821178 | 8 | 78093837 | A/C | 0.51 | 21102462 | AM |
| RBM6 | rs6762477 | 3 | 50093209 | G/A | 0.27 | 21102462 | AM |
| RXRG | rs466639 | 1 | 165394882 | A/G | 0.15 | 21102462 | AM |
| SEC16B | rs633715 | 1 | 177852580 | G/A | 0.1 | 21102462 | AM |
| SLC14A2 | rs2243803 | 18 | 42956672 | T/A | 0.86 | 21102462 | AM |
| SPOCK | rs13357391 | 5 | 136441082 | G/A | 0.12 | 19282985 | AM |
| SPOCK | rs1859345 | 5 | 136447420 | G/A | 0.13 | 19282985 | AM |
| TMEM18 | rs2947411 | 2 | 614168 | A/G | 0.23 | 21102462 | AM |
| TRIM66 | rs4929923 | 11 | 8639200 | A/G | 0.47 | 21102462 | AM |

| | | | | | | | |
|---|---|---|---|---|---|---|---|
| VGLL3 | rs7642134 | 3 | 86916882 | A/G | 0.56 | 21102462 | AM |
| ARHGEF7 | rs7333181 | 13 | 112221297 | A/G | 0.13 | 19448619 | ANM |
| BRSK1 | rs1172822 | 19 | 55819845 | A/G | 0.37 | 19448619 | ANM |
| GCM2 | rs2153157 | 6 | 10897488 | A/G | 0.44 | 19448621 | ANM |
| MCM8 | rs236114 | 20 | 5935385 | A/G | 0.22 | 19448619 | ANM |
| Intergenic | rs11889862 | 2 | 150697148 | A/G | 0.2 | 19448619 | ANM |
| Intergenic | rs17153527 | 7 | 106495809 | G/A | 0.13 | 19448619 | ANM |
| Intergenic | rs2151145 | 9 | 82345881 | G/A | 0.21 | 19448619 | ANM |
| Intergenic | rs2326679 | 20 | 6035228 | G/A | 0.26 | 19448619 | ANM |
| Intergenic | rs4397868 | 11 | 133566985 | G/A | 0.15 | 19448619 | ANM |
| Intergenic | rs4843747 | 16 | 87991051 | A/C | 0.17 | 19448619 | ANM |
| Intergenic | rs4906172 | 14 | 102454933 | A/C | 0.22 | 19448619 | ANM |
| Intergenic | rs494620 | 6 | 31838713 | G/A | 0.49 | 19448619 | ANM |
| Intergenic | rs4955755 | 3 | 170494409 | G/A | 0.3 | 19448619 | ANM |
| Intergenic | rs6468442 | 8 | 37686749 | A/G | 0.25 | 19448619 | ANM |
| TRMT11 | rs1361108 | 6 | 126767600 | A/G | 0.48 | 19448619 | ANM |
| TRMT6 | rs16991615 | 20 | 5948227 | A/G | 0.08 | 19448621 | ANM |
