# Supplementary material for: Genetic variations, reproductive aging, and breast cancer risk in African American and European American women: The Women's Circle of Health Study
Source: PLoS One. 2017 Oct 26;12(10):e0187205. doi: 10.1371/journal.pone.0187205 (PMC5658184; doi:10.1371/journal.pone.0187205)
Supplement: S2 Table — (PDF) [file pone.0187205.s002.pdf]

S2 Table. Descriptive characteristics of the Women's Circle of Health Study

Characteristics	African American			European-American		
	Case (n=621)	Controls (n=744)	P-value*	Case (n=658)	Control (n=649)	P-value*
Age, years, mean (SD)	51.4 (10.4)	48.6 (9.4)	<.0001	52.1 (10.1)	49.7 (8.7)	<.0001
Current body mass index, kg/m², mean (SD)	31.1 (6.7)	31.9 (7.8)	0.04	27.2 (6.6)	27.3 (7.0)	0.83
% European Ancestry, mean (SD)	13.7 (0.16)	13.8 (0.14)	0.92	96.8 (7.9)	98.5 (3.7)	<.0001
Education, n(%)			0.11			<.0001
High school or less	280 (45.1)	295 (39.7)		135 (20.5)	73 (11.2)	
College	274 (44.1)	354 (47.6)		345 (52.4)	327 (50.4)	
Post-graduate degree	67 (10.8)	95 (12.8)		178 (27.1)	249 (38.4)	
Smoking status, n (%)			<.0001			0.293
Never smoker	389 (62.6)	425 (57.1)		340 (51.8)	361 (55.6)	
Former smoker	147 (23.7)	141 (19.0)		239 (36.4)	210 (32.4)	
Current smoker	85 (13.7)	178 (23.9)		78 (11.9)	78 (12.0)	
Family history of breast cancer, n(%)			0.15			0.0006
Yes	89 (14.3)	87 (11.7)		161 (24.5)	109 (16.8)	
No	532 (85.7)	657 (88.3)		497 (75.5)	540 (83.2)	
Age at menarche, years, mean (SD)	12.5 (1.8)	12.5 (1.8)	0.39	12.5 (1.5)	12.6 (1.6)	0.32
Age at menarche, n (%)			0.85			0.21
≤ 12 years of age	316 (50.9)	382 (51.4)		331 (50.7)	304 (47.2)	
> 12 years of age	305 (49.1)	361 (48.6)		322 (49.3)	340 (52.8)	
Menopause status, n (%)			0.04			0.32
Premenopausal	309 (49.8)	412 (55.4)		343 (52.1)	356 (54.8)	
Postmenopausal	312 (50.2)	332 (44.6)		315 (47.9)	293 (45.2)	
Age at menopause, years, mean (SD)	49.7 (4.8)	49.1 (4.5)	0.09	50.4 (4.3)	50.0 (4.1)	0.29
Age at menopause, n (%)			0.32			0.75
≤ 50 years of age	199 (65.5)	227 (69.2)		165 (53.6)	158 (54.9)	
> 50 years of age	105 (34.5)	101 (30.8)		143 (46.4)	130 (45.1)	
Total reproductive life span, years, mean (SD)	37.9 (5.4)	36.6 (5.5)	0.006	38.1 (4.6)	37.4 (4.0)	0.016
Net reproductive life span, years, mean (SD)	35.1 (6.0)	34.2 (6.1)	0.14	36.5 (4.8)	35.4 (4.6)	0.007

Footnote: * P-value from student's T-test or chi-square test. Abbreviation: SD, standard deviation.
